# Impact of tissue inhibitor of metalloproteinases-3 genetic variants on clinicopathological characteristics of urothelial cell carcinoma

**DOI:** 10.7150/jca.81083

**Published:** 2023-01-22

**Authors:** Wei-Chun Weng, Ming-Hong Hsieh, Hui-Ling Chiou, Chia-Yi Lee, Chih-Hsin Tang, Lun-Ching Chang, Shian-Shiang Wang, Shun-Fa Yang

**Affiliations:** 1 Division of Urology, Department of Surgery, Tungs' Taichung MetroHarbor Hospital, Taichung, Taiwan; 2 Department of Nursing, Jen-Teh Junior College of Medicine, Nursing and Management, Miaoli, Taiwan; 3 School of Medicine, Chung Shan Medical University, Taichung, Taiwan; 4 Department of Psychiatry, Chung Shan Medical University Hospital, Taichung, Taiwan; 5 School of Medical Laboratory and Biotechnology, Chung Shan Medical University, Taichung, Taiwan; 6 Department of Ophthalmology, Nobel Eye Institute, Taipei, Taiwan; 7 School of Medicine, China Medical University, Taichung, Taiwan; 8 Chinese Medicine Research Center, China Medical University, Taichung, Taiwan; 9 Department of Medical Laboratory Science and Biotechnology, College of Medical and Health Science, Asia University, Taichung, Taiwan; 10 Department of Mathematical Sciences, Florida Atlantic University, Boca Raton, FL, USA; 11 Division of Urology, Department of Surgery, Taichung Veterans General Hospital, Taichung, Taiwan; 12 Department of Applied Chemistry, National Chi Nan University, Nantou, Taiwan; 13 Institute of Medicine, Chung Shan Medical University, Taichung, Taiwan; 14 Department of Medical Research, Chung Shan Medical University Hospital, Taichung, Taiwan

**Keywords:** single nucleotide polymorphism, urothelial cell carcinoma, tissue inhibitor of metalloproteinases-3, tobacco, tumor stage

## Abstract

To investigate the distribution of single nucleotide polymorphism (SNP) of tissue inhibitor of metalloproteinases-3 (*TIMP-3*) in patients with/without urothelial cell carcinoma (UCC), three loci of *TIMP-3* SNPs (rs9862 C/T, rs9619311 T/C, rs11547635 C/T) were genotyped via TaqMan allelic discrimination for 424 UCC patients and 848 non-UCC participants. Furthermore, the TIMP-3 mRNA expression and its correlation with clinical characters of urothelial bladder carcinoma was analyzed using The Cancer Genome Atlas database (TCGA). The distribution of all 3 studied SNPs of *TIMP-3* was insignificantly different between the UCC and non-UCC groups. However, significantly lower tumor T status was found in TIMP-3 SNP rs9862 CT + TT variant than the wild type (OR: 0.515, 95% CI: 0.289-0.917, P = 0.023). Moreover, the muscle invasive tumor type was significantly correlated to the *TIMP-3* SNP rs9619311 TC + CC variant in the non-smoker subgroup (OR: 2.149, 95% CI: 1.143-4.039, P = 0.016). With the TIMP-3 expression data provided in TCGA, significantly higher TIMP-3 mRNA expression was observed in UCC with high tumor stage (P < 0.0001), high tumor T status (P < 0.0001) and high lymph node status (P = 0.0005). In conclusions, *TIMP-3* SNP rs9862 variant is associated with lower tumor T status of UCC while *TIMP-3* SNP rs9619311 variant is correlated to muscle invasive UCC development in non-smoker.

## Introduction

The urothelial cell carcinoma (UCC) is a common neoplasm with an annual incidence rates above 3 per 100,000 person-years in the eastern Asian region 1. In the advanced form, the treatment options of UCC including surgery, platinum-based chemotherapy, immunotherapy and targeted therapy 2, 3. About the risk factor of UCC, the tobacco consumption is the most important risk factor which may account for nearly half of UCC cases 4. Also, the occupational exposure to substances like aromatic amines is a predictor for UCC development 5.

About the genetic aspect, several genes and their products are related to the development of UCC. The matrix metalloproteinases are a well-established genetic risk factor for the UCC occurrence especially for the matrix metalloproteinases-2 and matrix metalloproteinases-9 6. In addition, the expression of CCNA1 was significantly higher in the urine of UCC patients compared to the control group 7. On the other hand, the single nucleotide polymorphism (SNP) of certain genes would influence the incidence or characters of UCC. For instance, certain SNP of *AURKA* including rs2064863 and rs6024836 made a prominent influence on the clinical characteristics of UCC which mainly retarded the tumor progression 8. Besides, the patients with *HMGB1* rs1045411T allele were under a lower risk of UCC development 9. Accordingly, other gene or SNP may also affect the clinical status of UCC.

The tissue inhibitor of metalloproteinases-3 (TIMP-3) can alter the activity of the matrix metalloproteinases family and a higher concentration of TIMP-3 was observed in the patients with malignancies compared to those without such lesions 10-13. In previous researches, the TIMP-3 and its genetic polymorphism would affect the clinical features of oral cancer and lung adenocarcinoma 14, 15. However, there was rare research discussing the relationship between the genetic variant of *TIMP-3* and UCC. In previous studies, both oral cancer and UCC are associated with epithelial agent 16-20, plus the TIMP-3 can alter the activity of the matrix metalloproteinases which is related to the UCC development 6. Consequently, *TIMP-3* and its SNP variant may influence UCC progression which needs further investigation.

The purpose of current study is to evaluate the correlation between the SNP of *TIMP-3* and clinicopathological characters of UCC in a Taiwanese population. In addition, the results of urothelial bladder carcinoma from The Cancer Genome Atlas database were included and discussed.

## Materials and Methods

### Subject selection

This study was executed in Taichung Veterans General Hospital. Those who diagnosed with UCC in the Taichung Veterans General Hospital were selected and a total of 424 patients were included between Jan 2010 and Dec 2015 in the study group. Besides, subjects with history of cancer of any sites were excluded from the control group. The demographic data included age, gender, tobacco consumption history of these patients was taken from the medical document. The Tumor, Node, Metastasis (TNM) status, tumor stages were classified according to the American Joint Committee on Cancer. Patients would be dropped out from the current study of the blood samples degraded before the genetic variants analyses. The content of our study was adhered to declaration of Helsinki in 1964 and associated amendments. The Institutional Review Boards of Taichung Veterans General Hospital also approved our study (Project code: no. CF11094; 27 July 2011). A written informed consent was obtained from each participant after explaining the details of our study. For the polymorphism of *TIMP-3*, venous blood sample was taken and then preserved in the ethylene-diaminetetraacetic acid-containing tubes. The blood samples were then centrifuged and put in our laboratory refrigerator at -80 degree Celsius for our analyses.

### Genomic DNA extraction and analyze *TIMP-3* SNP via Real-Time PCR

Three SNPs of *TIMP-3* including rs9862 (C/T), rs9619311 (T/C), rs11547635 (C/T) were picked out since our previous experience showed the effect of these SNPs on the oral cancer 15. The genotyping procedure used in our study was similar as our previous research 21-23. The genome was firstly taken from leukocytes of blood sample via the QIAamp DNA kits (Qiagen, Valencia, Valencia, CA, USA), and all the procedures with QIAamp DNA kits was adhered to the manufacture's guideline. We preserved theses isolated DNA in refrigerators under -20 degree Celsius. In the next step, the three *TIMP-3* genetic polymorphisms we selected were analyzed with the use of ABI StepOne Real-Time PCR System (Applied Biosystems, Foster City, California). After all the procedures, the genetic polymorphisms about the three *TIMP-3* SNPs were analyzed via TaqMan assay technique and SDS version 3.0 software (Applied Biosystems) to augment the completeness of Real-Time PCR in our study.

### Bioinformatics analysis of TIMP-3 expression

For the potential association between TIMP-3 expression and clinical status of UCC, we use the data of urothelial bladder carcinoma obtained from The Cancer Genome Atlas (TCGA) to analyze this issue 24-26. In this part of analysis, urothelial bladder carcinoma was divided into different subgroup according to the tumor stage and TNM statuses, then the mRNA level of TIMP-3 was compared between the subgroups.

### Statistical analysis

The SAS version 9.4 (SAS Institute Inc, Cary, NC, USA) was used for the statistical analyses in the current study. Descriptive analysis including mean, standard deviation (SD) and percentage was used to showed the demography and laboratory data between the non-UCC and UCC groups. The student's t test or chi-squared test was used to compare different parameters between control group and patients with UCC. Then the logistic regression models were used to produce the odds ratio (OR) and associated 95% confidence interval (CI) about the polymorphism distribution between the non-UCC and UCC population. Moreover, the adjusted odds ratio (AOR) with 95% CI between the two groups was calculated via multiple logistic regression models after adjusting age, gender and tobacco consumption. For the subgroup analyses in the UCC population, the distribution frequencies between the different genotypes of *TIMP-3* SNP rs9862 as well as rs9619311 and the clinical condition of UCC were presented as OR with 95% CI. Further, we divided the UCC population into non-smoker and smoker, and the distribution frequency between *TIMP-3* SNP rs9619311 and clinicopathological characters of UCC was analyzed and then produced the AOR with 95% CI. The statistically significant level was set as P < 0.05 in the current study and those with P value lesser than 0.001 was presented as P < 0.001.

## Results

### Basic characters between the non-UCC and UCC groups

The demography of the non-UCC and UCC groups are shown in Table 1. The mean age in the non-UCC group was 57.09 ± 10.04 years which was significant younger than that in the UCC group (68.58 ± 11.84, P < 0.001), while the gender and tobacco consumption distribution did not differ between the two groups (both P > 0.05). The tumor feature of the UCC group including tumor stage, TNM status and histopathologic grading are also available in Table 1.

### Distribution frequencies of TIMP-3 SNPs between non-UCC and UCC groups

The genotype distribution of *TIMP-3* SNPs between the non-UCC and UCC population are presented in the Table 2. Both the *TIMP-3* SNP rs9862 CT+TT and *TIMP-3* SNP rs9619311 TC+CC were numerically higher in the UCC group than the non-UCC group, while the *TIMP-3* SNP rs11547635 CT+TT was numerically lower in the UCC group than the non-UCC group. Nevertheless, none of these values demonstrated significant difference between the UCC and non-UCC group regarding both the OR and AOR which adjusting age, gender and tobacco consumption (all P > 0.05) (Table 2).

### Subgroup Analyses of TIMP-3 SNPs Distribution in the UCC group

In the subgroup analyses, the relationship between the clinical status of UCC and the *TIMP-3* SNP rs9862 genotype is shown in Table 3. The *TIMP-3* SNP rs9862 CT + TT variant owned a significantly lower tumor T status than the SNP rs9862 CC wild type (OR: 0.515, 95% CI: 0.289-0.917, P = 0.023) while the SNP rs9862 variant and SNP rs9862 wild type showed no difference in other tumor conditions (all P > 0.05) (Table 3). About the *TIMP-3* rs9619311 genotype frequencies and the clinical characters of UCC, a similar tumor status was found in each parameter between the *TIMP-3* rs9619311 TC + CC variant and *TIMP-3* rs9619311 TT wild type (all P > 0.05) (Table 4). After dividing the UCC population into the non-smoker and smoker, the presence of muscle invasive tumor type was significantly correlated to the *TIMP-3* SNP rs9619311 TC + CC variant in the non-smoker subgroup (OR: 2.149, 95% CI: 1.143-4.039, P = 0.016) (Table 5). On the other hand, the *TIMP-3* SNP rs9619311 genotypes did not correlate to the change of UCC clinopathological characters in the smoker group (all P > 0.05) (Table 5).

### TIMP-3 mRNA expression in the urothelial bladder carcinoma from TCGA dataset

**Figure 1:**
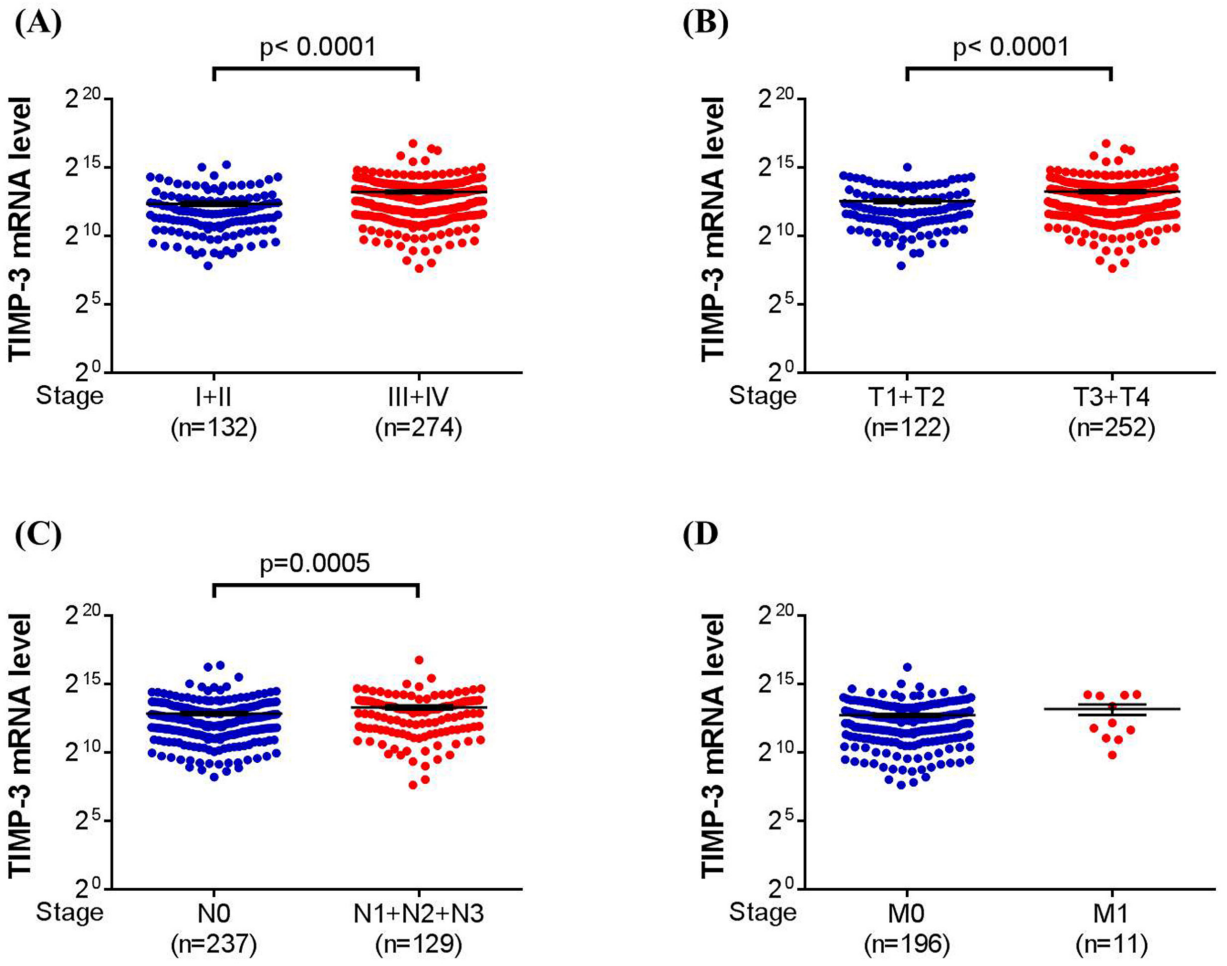
The TIMP-3 expression in the urothelial bladder carcinoma with different grade according to The Cancer Genome Atlas database. (A) The expression of TIMP-3 mRNA in different tumor stages. (B) The expression of TIMP-3 mRNA in different tumor T statuses. (C) The expression of TIMP-3 mRNA in different lymph node statuses. (D) The expression of TIMP-3 mRNA in different metastasis statuses

About the TIMP-3 expression in the database from TCGA, we categorized the urothelial bladder carcinoma into low tumor stage (stage I and II) and high tumor stage (stage III and IV), low tumor T status (T1 and T2) and high tumor T status (T3 and T4), no lymph node status (N0) and lymph node status (N1 to N3), and no metastasis (M0) and metastasis (M1). After the analyses, a significantly higher TIMP-3 mRNA level was found in high tumor stage (P < 0.0001), high tumor T status (P < 0.0001) and high lymph node status (P = 0.0005) (Figure 1A-1C). Still, the TIMP-3 mRNA level between no metastasis and metastasis form of urothelial bladder carcinoma was nearly identical (Figure 1D).

## Discussion

In our study, the *TIMP-3* SNP rs9862 variant is associated with lower tumor T status in patients with UCC. Moreover, the *TIMP-3* SNP rs9619311 variant is correlated to higher tumor stage in the non-smoker population who diagnosed with UCC. Besides, the data from TCGA demonstrated that the TIMP-3 mRNA levels showed significant relationship to higher tumor stage, tumor T status and lymph node status in urothelial bladder carcinoma.

Many gene and related polymorphisms would influence the clinical course of UCC in preceding researches. The endothelial nitric oxide synthase rs1799983 GT + TT variants own higher risk of developing large tumor 27. And the *RAGE* gene and its polymorphism have been demonstrated to cause high UCC incidence and worse disease-specific survival 28. Other genetic predictors of UCC include the mutation on TP53/MDM2, RAS, FGFR3, hyper-mutated and triple negative transform 29. On the other hand, there is also genetic protector for the UCC, in which low level of growth arrest-specific 5 expression in female with bladder urothelial carcinoma showed poorer overall survival rate 30. About the TIMP-3, this gene is correlated to various diseases including several malignancies 31, 32. In previous studies, the TIMP-3 would cause higher possibility of cardiovascular diseases development such as myocardial infarction and coronary arterial plaque 33, 34. For the field of neoplasm, the TIMP-3 and its polymorphism showed significant association to the colorectal cancer and prostate cancer 35, 36. Moreover, the character of TIMP-3 that can serve as tumor progression predictor let TIMP-3 own the potentiality to become a target for cancer therapy 37. Because the TIMP-3 illustrated such characters on several tumors, and considering the effect of matrix metalloproteinases on UCC 6, we speculate that the genotype of TIMP-3 may affect the clinical condition of UCC, whether a predictor or protector. Our hypothesis was supported by the results of the current study at least to some degrees.

For the *TIMP-3* SNP variants and the clinicopathological characteristics of UCC, none of the three* TIMP-3* SNP variants analyzed in the current study showed significant difference of distribution frequencies between the non-UCC and UCC population. However, the *TIMP-3* SNP rs9862 CT + TT genotype is associated with lower tumor T status in the UCC patients. About the percentage aspect, 76.6 percent of *TIMP-3* SNP rs9862 CT + TT genotype showed advanced tumor T status while 86.4 percent of *TIMP-3* SNP rs9862 CC genotype illustrated advanced tumor T status. In previous researches, the TIMP-3 has both tumorigenic and anti-tumorigenic properties 38, 39. Accordingly, the genotype of *TIMP-3* SNP rs9862 CT + TT may be a protector for the UCC in general population.

Concerning the subgroup analyses in the current study dependent on the existence of tobacco consumption, the UCC individuals who never smoke would experience higher tumor stage of the *TIMP-3* SNP rs9619311 variant was existed. This is a relative novel finding in the field of UCC to our knowledge. The muscle invasive tumor is a high-risk form of UCC which needs more complicated therapy than the non-muscle invasive type which may be treated with a curative intent 40, 41. The previous study showed that the five years survival rate of muscle invasive tumor was around 40 percent 42, which was significantly lower than that in the non-muscle invasive type 43. Consequently, to find the patients who own higher risk of muscle invasive tumor development should be emphasized. Our study demonstrated that the non-smoker with *TIMP-3* SNP rs9619311 TC + CC genotype may be under higher risk of muscle invasive tumor development, thus these patients may be suitable to receive aggressive therapy at the early stage while further experiments are needed to support this concept.

In the TCGA analysis, the TIMP-3 showed higher level of mRNA expression in the UCC with advanced tumor stage, tumor T status and lymph node status. TCGA database enrolled considerable patients with urothelial bladder carcinoma and related literature has been published before 44. Because of our study design, we did not analyze the quantity of TIMP-3 mRNA expression, but the TCGA database can compensate this shortness in our study. The findings of TCGA data, combined with the results of our patients, illustrated that the TIMP-3 could lead to the higher incidence of advanced UCC development while the* TIMP-3* SNP genotypes would alter this condition. This may further highlight the prominent influence of SNP on a tumor-aggregating gene. On the other hand, the existence of metastasis did not influence by the TIMP-3 mRNA expression in TCGA database, and we also found that none of *TIMP-3* SNP is associated with the ratio of metastasis. The reasons that TIMP-3 has minimal effect on the UCC metastasis need further evaluation.

In conclusion, individuals with UCC are associated with lower level of tumor T status under the presence of *TIMP-3* SNP rs9862 variant. Furthermore, the *TIMP-3* SNP rs9619311 variant may lead to higher tumor stage of UCC in the non-smoker population, which is in accordance with the oncogenic effect of TIMP-3 for UCC according to the result of TCGA analysis. Consequently, the presence of *TIMP-3* SNP rs9619311 variant might be screened for patients with UCC to find those with high possibility of muscle invasive tumor. Further population-based prospective study to survey whether the SNP variant of *TIMP-3* would affect the therapeutic outcome and survival rate of UCC is mandatory.

## Figures and Tables

**Table 1:** The distributions of demographical characteristics in 848 controls and 424 patients with UCC.

Variable	Non-UCC (N=848)n (%)	UCC (N=424)n (%)	P value
Age (yrs)			
Mean ± SD	57.09 ± 10.04	68.58 ± 11.84	<0.001
Gender			0.320
Male	554 (65.3%)	265 (62.5%)	
Female	294 (34.7%)	159 (37.5%)	
Tobacco consumption			0.151
No	558 (65.8%)	296 (69.8%)	
Yes	290 (34.2%)	128 (30.2%)	
Stage			
Non muscle invasive tumor (pTa-pT1)		231 (54.5%)	
Muscle invasive tumor (pT2-pT4)		193 (45.5%)	
Tumor T status			
Ta		87 (20.5%)	
T1-T4		337 (79.5%)	
Lymph node status			
N0		374 (88.2%)	
N1+N2		50 (11.8%)	
Metastasis			
M0		411 (96.9%)	
M1		13 (3.1%)	
Histopathologic grading			
Low grade		51 (12.0%)	
High grade		373 (88.0%)	

N: number; SD: standard deviation

**Table 2:** Genotype Distributions of TIMP-3 Gene Polymorphisms in 848 Controls and 424 Patients with UCC.

Variable	Non-UCC (N=848) n (%)	UCC (N=424) n (%)	OR (95% CI)	AOR (95% CI)
**rs9862**				
CC	293 (34.6%)	125 (29.5%)	1.000 (reference)	1.000 (reference)
CT	393 (46.3%)	219 (51.7%)	1.306 (0.997-1.706)	1.256 (0.926-1.704)
TT	162 (19.1%)	80 (18.8%)	1.158 (0.824-1.626)	1.100 (0.747-1.619)
CT+TT	555 (65.4%)	299 (70.5%)	1.124 (0.991-1.275)	1.100 (0.952-1.271)
**rs9619311**				
TT	702 (82.8%)	346 (81.6%)	1.000	1.000 (reference)
TC	135 (15.9%)	76 (17.9%)	1.142 (0.838-1.556)	1.222 (0.859-1.739)
CC	11 (1.3%)	2 (0.5%)	0.369 (0.081-1.673)	0.490 (0.098-2.439)
TC+CC	146 (17.2%)	78 (18.4%)	1.041 (0.895-1.212)	1.082 (0.910-1.286)
**rs11547635**				
CC	374 (44.1%)	192 (45.3%)	1.000 (reference)	1.000 (reference)
CT	374 (44.1%)	189 (44.6%)	0.984 (0.769-1.260)	0.949 (0.716-1.258)
TT	100 (11.8%)	43 (10.1%)	0.838 (0.563-1.246)	0.700 (0.444-1.101)
CT+TT	474 (55.9%)	232 (54.7%)	0.976 (0.868-1.098)	0.945 (0.827-1.080)

N: number OR: odds ratio AOR: adjusted odds ratio with their 95% confidence intervals were estimated by multiple logistic regression models after controlling for age, gender and tobacco consumption. CI: confidence interval

**Table 3:** Distribution frequency of the clinical status and TIMP-3 rs9862 genotype frequencies in 424 UCC patients.

Variable	TIMP-3 (rs9862)			
	CC (%) (n=125)	CT + TT (%) (n=299)	OR (95% CI)	P value
**Stage**				
Non muscle invasive tumor (pTa-pT1)	63 (50.4%)	168 (56.2%)	1.000 (reference)	0.275
Muscle invasive tumor (pT2-pT4)	62 (49.6%)	131 (43.8%)	0.792 (0.521-1.204)	
**Tumor T status**				
Ta	17 (13.6%)	70 (23.4%)	1.000 (reference)	0.023*
T1-T4	108 (86.4%)	229 (76.6%)	**0.515 (0.289-0.917)**	
**Lymph node status**				
N0	108 (86.4%)	266 (89.0%)	1.000 (reference)	0.456
N1+N2	17 (13.6%)	33 (11.0%)	0.788 (0.421-1.475)	
**Metastasis**				
M0	123 (98.4%)	288 (96.3%)	1.000 (reference)	0.258
M1	2 (1.6%)	11 (3.7%)	2.349 (0.513-10.755)	
**Histopathologic grading**				
Low grade	15 (12.0%)	36 (12.0%)	1.000 (reference)	0.991
High grade	110 (88.0%)	263 (88.0%)	0.996 (0.524-1.893)	

N: number OR: odds ratio * denotes significant difference between the two groups

**Table 4:** Distribution frequency of the clinical status and TIMP-3 rs9619311 genotype frequencies in 424 UCC patients.

Variable	TIMP-3 (rs9619311)			
	TT (%) (n=346)	TC + CC (%) (n=78)	OR (95% CI)	P value
**Stage**				
Non muscle invasive tumor (pTa-pT1)	195 (56.4%)	36 (46.2%)	1.000 (reference)	0.102
Muscle invasive tumor (pT2-pT4)	151 (43.6%)	42 (53.8%)	1.507 (0.920-2.467)	
**Tumor T status**				
Ta	74 (21.4%)	13 (16.7%)	1.000 (reference)	0.351
T1-T4	272 (78.6%)	65 (83.3%)	1.360 (0.711-2.602)	
**Lymph node status**				
N0	306 (88.4%)	68 (87.2%)	1.000 (reference)	0.755
N1+N2	40 (11.6%)	10 (12.8%)	1.125 (0.536-2.361)	
**Metastasis**				
M0	336 (97.1%)	75 (96.2%)	1.000 (reference)	0.658
M1	10 (2.9%)	3 (3.8%)	1.344 (0.361-5.002)	
**Histopathologic grading**				
Low grade	42 (12.1%)	9 (11.5%)	1.000 (reference)	0.883
High grade	304 (87.9%)	69 (88.5%)	1.059 (0.492-2.278)	

N: number OR: odds ratio

**Table 5:** Distribution frequency of the clinical status and TIMP-3 rs9619311 genotype frequencies in 424 UCC patients with cigarette smoking status.

Variable	TIMP-3 (rs9619311)					
	Non-Smoker (N=296)			Smoker (N=128)		
	TT (%) (n=248)	TC + CC (%) (n=48)	P value	TT (%) (n=98)	TC + CC (%) (n=30)	P value
**Stage**						
Non muscle invasive tumor (pTa-pT1)	145 (58.5%)	19 (39.6%)	**0.016^a^**	50 (51.0%)	17 (56.7%)	0.588
Muscle invasive tumor (pT2-pT4)	103 (41.5%)	29 (60.4%)		48 (49.0%)	13 (43.3%)	
**Tumor T status**						
Ta	51 (20.6%)	8 (16.7%)	0.536	23 (23.5%)	5 (16.7%)	0.430
T1-T4	197 (79.4%)	40 (83.3%)		75 (76.5%)	25 (83.3%)	
**Lymph node status**						
N0	222 (89.5%)	42 (87.5%)	0.681	84 (85.7%)	26 (86.7%)	0.896
N1+N2	26 (10.5%)	6 (12.5%)		14 (14.3%)	4 (13.3%)	
**Metastasis**						
M0	245 (98.8%)	46 (95.8%)	0.146	91 (92.9%)	29 (96.7%)	0.451
M1	3 (1.2%)	2 (4.2%)		7 (7.1%)	1 (3.3%)	
**Histopathologic grading**						
Low grade	32 (12.9%)	3 (6.3%)	0.191	10 (10.2%)	6 (20.0%)	0.156
High grade	216 (87.1%)	45 (93.8%)		88 (89.8%)	24 (80.0%)	

N: number OR: odds ratio ^a^OR and 95CI: 2.149 (1.143-4.039)
